# Thalamic *Htr3a* Gene Expression in Hemi-Parkinsonian Rats Subjected to Persistent Pain

**DOI:** 10.3390/brainsci16101065

**Published:** 2026-10-03

**Authors:** José Luis Cortes-Altamirano, Alfonso Alfaro-Rodríguez, Samuel Reyes-Long, Francisco Ulises Ortiz-Moreno, Herlinda Bonilla-Jaime, Angelica González-Maciel, Beatriz Pérez-Guille, Rosa Eugenia Soriano-Rosales, Rafael Reynoso-Robles, David Calderon-Guzman, Hugo Juaréz-Olguín

**Affiliations:** 1 Division of Basic Neurosciences, National Institute of Rehabilitation-LGII, Mexico City 14389, Mexico; drjlcortesaltamirano@gmail.com (J.L.C.-A.); sam.long27@gmail.com (S.R.-L.); omfraul@gmail.com (F.U.O.-M.); 2 Department of Chiropractic, Universidad Estatal del Valle de Ecatepec, Ecatepec de Morelos 55210, Mexico; 3 CONAHCyT National Laboratory in Artificial Intelligence and Data Science (LNC-IACD), Ecatepec de Morelos 55210, Mexico; 4 Department of Reproductive Biology, Universidad Autónoma Metropolitana, Mexico City 09340, Mexico; lindabon35@gmail.com; 5 Laboratory of Cellular and Tissue Morphology, National Institute of Pediatrics, Ministry of Health, Mexico City 04300, Mexico; agonzalezmaciel@yahoo.com (A.G.-M.); reynosoraf@yahoo.com (R.R.-R.); 6 Center for Translational Research, National Institute of Pediatrics, Mexico City 04300, Mexico; bettyepg@yahoo.com (B.P.-G.); resr62@yahoo.com.mx (R.E.S.-R.); 7 Laboratory of Neurosciences, National Institute of Pediatrics, Mexico City 04530, Mexico; davidcaguama91@gmail.com; 8 Department of Pharmacology, Faculty of Medicine, Universidad Nacional Autónoma de Mexico, Mexico City 04510, Mexico; juarezol@yahoo.com

**Keywords:** Parkinson’s disease, pain, 5-HT3A receptor, thalamus, serotonin

## Abstract

**Highlights:**

**What are the main findings?**

- Unilateral 6-OHDA lesion is associated with altered serotonergic gene regulation in the thalamus.
- Thalamic *Htr3a* mRNA expression decreased bilaterally following a 6-OHDA lesion in a persistent pain model.

**What are the implications of the main findings?**

- The ipsilateral thalamus showed a significantly greater *Htr3a* transcript downregulation than the contralateral hemisphere.
- The findings suggest region-dependent transcriptional remodeling of the *Htr3a* gene during hemi-Parkinsonian persistent pain.

**Abstract:**

**Background**: Pain is a frequent and debilitating non-motor symptom of Parkinson’s disease; however, its central neurochemical mechanisms remain incompletely understood. Serotonin modulates nociceptive processing, and the 5-HT3A receptor has been implicated in excitatory signaling and central sensitization. However, thalamic *Htr3a* transcript regulation during persistent pain associated with dopaminergic injury remains unclear. **Objective**: To evaluate thalamic *Htr3a* mRNA expression in 6-OHDA hemi-Parkinsonian rats subjected to formalin-induced persistent pain. **Methods**: Male Wistar rats were assigned to six groups (n = 6/group): Saline, Formalin, SHAM + Saline, SHAM + Formalin, 6-OHDA + Saline, and 6-OHDA + Formalin. 6-OHDA lesions were validated via apomorphine-induced rotations (>7 turns/min). Nociceptive behavior (flinches) was recorded over 60 min post-formalin injection. Bilateral thalamic *Htr3a* mRNA expression was quantified by RT-qPCR using Rpl27 as a validated reference gene. **Results**: 6-OHDA + Formalin rats exhibited a significant increase in persistent-phase nociceptive behavior compared to controls (*p* < 0.05). *Htr3a* mRNA expression showed a significant bilateral reduction in 6-OHDA-lesioned rats compared to controls (*p* < 0.05), with the decrease being significantly more severe in the ipsilateral thalamus (*p* < 0.001). **Conclusions**: Unilateral 6-OHDA lesion is associated with a region-dependent reduction in thalamic *Htr3a* mRNA expression during persistent pain. These results demonstrate transcriptional adaptations of the thalamic serotonergic system in experimental parkinsonian persistent pain.

## 1. Introduction

Parkinson’s disease (PD) is the second most common neurodegenerative disorder worldwide and is characterized by the progressive loss of dopaminergic neurons in the substantia nigra *pars compacta* [1]. Although traditionally considered a movement disorder because of cardinal symptoms such as bradykinesia, rigidity, resting tremor, and postural instability, PD is now recognized to have non-motor symptoms that significantly contribute to disease progression and deterioration in patients’ quality of life [2,3,4]. Among these manifestations, pain is one of the most prevalent, debilitating, and least understood symptoms from a pathophysiological perspective [5].

It is estimated that between 40% and 80% of patients with PD experience pain throughout the course of the disease, which may manifest even before the onset of classic motor symptoms [6]. It has been suggested that alterations in central nociceptive pathways play a significant role in developing this symptom, as dopaminergic neurodegeneration affects brain structures involved in the perception, integration, and modulation of pain [7]. However, the neurochemical mechanisms responsible for the nociceptive hypersensitivity observed in PD have not yet been fully elucidated, limiting the development of therapeutic strategies specifically aimed at controlling the pain associated with this disease.

Serotonin (5-hydroxytryptamine, 5-HT) is a major neurotransmitter that modulates nociceptive processing at both central and peripheral levels [8]. Serotonergic neurons originating in the raphe nuclei project extensively to structures involved in pain perception and regulation, including the spinal cord, brainstem, thalamus, and various cortical regions [9]. Through its many receptor subtypes, serotonin can exert facilitatory or inhibitory effects on nociceptive transmission, depending on the anatomical region and pathophysiological context [10,11,12]. Several studies have shown that alterations in serotonergic neurotransmission contribute to the development and maintenance of persistent pain, and dysfunction of this system has also been widely documented in Parkinson’s disease [13,14,15]. Taken together, these findings suggest that serotonergic mechanisms may be involved in the alterations in nociceptive processing observed during dopaminergic neurodegeneration.

Among serotonergic receptors involved in pain modulation, the 5-HT3A receptor is notable because it is the only ionotropic serotonergic receptor described to date. Unlike G protein-coupled receptors, activation of the 5-HT3A receptor rapidly depolarizes neurons by opening cation channels, thereby promoting nociceptive signal transmission [16]. Experimental studies show that this receptor is involved in central sensitization, inflammatory pain, and neuropathic pain, contributing to persistent hypersensitivity. It has also been proposed that alterations in 5-HT3A-mediated signaling may modify neuronal excitability and sensory information integration in regions involved in pain processing [17,18].

Furthermore, the thalamus serves as a major relay and integration station for sensory and nociceptive information to the cerebral cortex, playing a fundamental role in pain perception and discrimination [19,20]. Several studies have shown that functional and neurochemical alterations in thalamic circuits contribute to the development of hypersensitivity and chronic pain and also play a role in the nonmotor symptoms observed in PD [21]. Because this structure receives extensive serotonergic innervation and is involved in nociceptive processing, changes in serotonergic receptor expression could alter pain-signal integration during dopaminergic neurodegeneration. However, the specific role of the 5-HT3A receptor in the thalamus under conditions of persistent pain associated with experimental parkinsonism remains poorly studied.

Despite growing evidence linking the serotonergic system to pain modulation, the specific role of the 5-HT3A receptor in the thalamus in persistent pain associated with Parkinson’s disease remains poorly understood. Because the thalamus is a major structure that integrates and relays nociceptive information to the cerebral cortex, alterations in serotonergic receptor expression could contribute to changes in pain processing observed during dopaminergic neurodegeneration. Therefore, this study aimed to evaluate thalamic *Htr3a* mRNA expression in hemi-parkinsonian rats subjected to a model of persistent formalin-induced pain. We hypothesized that the 6-OHDA lesion would modify thalamic *Htr3a* mRNA expression and potentiate formalin-evoked nociceptive behavior, particularly during the persistent phase.

## 2. Materials and Methods

A controlled, randomized experimental study was conducted. Male Wistar rats (*Rattus norvegicus*) with an average weight of 250 ± 15 g were used. The animals were housed in the conventional bioterium at the Instituto Nacional de Rehabilitación “Luis Guillermo Ibarra Ibarra”. All procedures were conducted in strict accordance with the technical specifications for the production, care, and use of laboratory animals and with the Official Mexican Norm (Norma Oficial Mexicana, NOM-062-ZOO-1999). In addition, the rats were treated in accordance with the guidelines “Care and Use of Experimental Animals” (Canadian Council on Animal Care). Due to the nature of the experiment, the guidelines set forth in the “Guidelines on Ethical Standards for Investigation of Experimental Pain in Animals” were followed as well. All procedures were approved by the Research Ethics Committee of the Instituto Nacional de Rehabilitación “Luis Guillermo Ibarra Ibarra”, with registration number INRLGII 98/22 SP-1, registration date: 27 June 2025.

### 2.1. Housing Conditions

The animals were housed in polypropylene cages lined with Aspen Shavings^®^, with a density of 0.66 m^2^ per animal. They were kept at a temperature of 22 ± 2 °C, relative humidity averaging 50 ± 10%, and were ventilated with 20 air changes per hour. The light cycle consisted of 12 h of light and 12 h of darkness. Water and a balanced pellet diet from Harlan Tekland Global Diets^®^ (USA) were provided I. The diet contained 18% crude protein, 3.5% crude fat, and 13% crude fiber.

### 2.2. Study Groups

A total of 42 male Wistar rats were initially allocated to the study. Six animals were excluded prior to final analysis due to failure to achieve the hemi-parkinsonian threshold in the apomorphine test (>7 turns/min, n = 4) or surgical complications (n = 2), resulting in a final sample size of n = 6 per group across six experimental groups. Thirty-six male Wistar rats (*Rattus norvegicus*) with an average weight of 250 ± 15 g were randomly assigned to six experimental groups (www.randomizer.org). Behavioral evaluations and RT-qPCR molecular analyses were performed by experimenters blinded to the treatment groups. Sample size (n = 6 per group) was determined based on previous literature using similar 6-OHDA and formalin pain paradigms; formal a priori power calculations were not performed. Group 1: Saline: Rats that did not undergo stereotactic surgery and received a subcutaneous saline injection in the dorsal region of the left hind limb. Nociceptive behavior was then assessed, and bilateral thalamic dissection was performed for molecular analysis. Group 2: Formalin: Rats that did not undergo stereotactic surgery and received a subcutaneous 1% formalin injection in the dorsal region of the left hind limb to induce persistent pain. Nociceptive behavior was then evaluated, and bilateral thalamic dissection was performed for molecular analysis. Group 3: SHAM + saline: Rats that underwent surgery and received a unilateral stereotactic injection of the vehicle (1% ascorbic acid) and, after the recovery period, a subcutaneous saline injection in the dorsal region of the left hind limb. Nociceptive behavior was then evaluated, and bilateral thalamic dissection was performed for molecular analysis. Group 4: SHAM + formalin: Rats that underwent surgery and received a unilateral stereotactic injection of the vehicle (1% ascorbic acid) and, after the recovery period, a subcutaneous 1% formalin injection in the dorsal region of the left hind limb to induce persistent pain. Nociceptive behavior was then evaluated, and bilateral thalamic dissection was performed for molecular analysis. Group 5: 6-OHDA + saline: Rats that received a unilateral stereotactic injection of 6-OHDA into the substantia nigra pars compacta. After a 20-day recovery period and verification of the hemi-Parkinsonian phenotype using the apomorphine test, they received a subcutaneous saline injection in the dorsal region of the left hind limb. Nociceptive behavior was then evaluated, and bilateral thalamic dissection was performed for molecular analysis. Group 6: 6-OHDA + formalin: Rats that received a unilateral stereotactic injection of 6-OHDA into the substantia nigra pars compacta. After a 20-day recovery period and verification of the hemi-Parkinsonian phenotype using the apomorphine test, the animals received a subcutaneous 1% formalin injection in the dorsal region of the left hind limb to induce persistent pain. Nociceptive behavior was then evaluated, and bilateral thalamic dissection was performed for molecular analysis.

### 2.3. Stereotactic Surgery

The rats were anesthetized with ketamine (70 mg/kg, Ketamin-Pet^®^ Aranda, Mexico) and xylazine (15 mg/kg, Procin^®^ PiSA, Mexico) via intraperitoneal (i.p.) injection. After 5 min, the level of anesthesia was assessed by compressing the distal third of the rat’s tail or pricking the plantar pads of its limbs until surgical anesthesia was achieved. Next, the incision site (between the frontal and occipital bones) was washed and shaved. The rats were secured to the stereotaxic apparatus (Stoeling Corp., Wood Dale, IL, USA), and their body temperature was monitored and regulated using a rat-specific temperature regulator (CMA/150). An iodine antiseptic solution was applied to the incision site, which was made with a No. 15 scalpel blade, creating a craniocaudal incision approximately 1.0 cm long. After exposing the skull, the intracranial suture known as the bregma was located (point 0). The coordinates were marked on the skull for administration into the substantia nigra *pars compacta* of the right hemisphere of the brain (Anteroposterior to bregma (AP) = −5.0 mm; Lateral (L) = −2.0 mm; Dorsoventral (DV) = −7.5 mm), according to the stereotactic atlas by Paxinos and Watson [22]. Next, the skull was drilled with a 0.7 mm diameter trephine using an electric handpiece (OSADA, XL-30W, Los Angeles, CA, USA). Then, 2 µL of 6-OHDA (6-Hidroxidopamina, Sigma-Aldrich, Darmstadt, Germany) at a concentration of 8 µg/µL, diluted in 1% ascorbic acid in Milli-Q water, was administered at a flow rate of 0.400 µL/min for 5 min. The incision was then closed with a 2/0 monofilament non-absorbable nylon suture and a needle with a longitudinally grooved shaft (SC-26), 26 mm, 3/8 circle, sharp reverse tip. The sutures were cleaned with iodine, and 2 mg/kg of meloxicam was administered subcutaneously (s.c.). All surgeries were performed between 8 a.m. and 12 p.m.

### 2.4. Recovery

After surgery, the rats were monitored for 6 h, with food and water available *ad libitum* in the cage to ensure easy access. A saline-moistened bandage was applied to the eyes to prevent drying. After the recovery period, the rats were allowed to rest for 20 days in the bioterium at the Instituto Nacional de Rehabilitación “Luis Guillermo Ibarra” under constant surveillance. During the first 3 days after surgery, 2 mg/kg of meloxicam was administered subcutaneously every 24 h.

### 2.5. Apomorphine Test

In a Plexiglas circle (20 × 24 cm) with mirrors arranged at a 45° angle to provide a complete view of the rats’ activity, sawdust from the rats’ usual cage was placed. The rats acclimated for 30 min before the test began. Apomorphine (0.5 mg/kg) was administered subcutaneously in the scapular region while the rat was immobilized with moderate restraint. After 5 min, the rat’s rotational behavior was observed, and the number of 360-degree turns was recorded over 40 min. Rats that exhibited more than 7 contralateral rotations per minute following apomorphine administration were considered to have hemi-Parkinsonism and were selected for further analysis [23].

### 2.6. Formalin Test

Formalin (50 µL of 1% solution) was injected subcutaneously into the plantar/dorsal surface of the left hind paw using a 26-gauge needle attached to a micro-syringe. Pain-related behaviors (hind paw flinches) were scored in real time by an observer blinded to group assignments and recorded for a second observer blinded as well, counting flinches during 1 min blocks every 5 min over 60 min. The acute phase (Phase I, 0–15 min) was defined based on direct chemical activation of peripheral nociceptors, while the persistent phase (Phase II, 15–60 min) reflects central sensitization and ongoing inflammatory cascades, as established in classical formalin test paradigms. All recordings were made between 9:00 a.m. and 1:00 p.m.

### 2.7. Dissection

Immediately after the formalin test, the animals were euthanized by guillotine decapitation performed by a single trained operator. The brain was quickly dissected and placed on a cold surface to minimize RNA degradation. Subsequently, the right and left thalami were dissected separately using the anatomical coordinates described in the Paxinos and Watson atlas [22]. We processed each hemisphere separately and stored it individually in 1.5 mL sterile tubes. The samples were immediately frozen at −80 °C until processed for RNA extraction and gene expression analysis by real-time PCR. The biological waste generated during the procedure was handled and disposed of in accordance with the Official Mexican Norm (NOM-087-ECOL-SSA1-2002) for biologically infectious hazardous waste.

### 2.8. Total RNA Extraction

Total RNA was extracted from each thalamic hemisphere separately using TRIzol Reagent^®^ (Invitrogen, Thermo Fisher Scientific, Waltham, MA, USA). Each sample was homogenized in 1 mL of TRIzol by repeated pipetting and then incubated at room temperature for 5 min. For each mL of TRIzol, add 100 μL of chloroform; shake vigorously for 15 s, then incubate at room temperature for 10 min. The samples were then centrifuged at 13,000× *g* for 15 min at 4 °C to separate the phases. The aqueous phase was carefully recovered and transferred to a clean tube, then the organic phase was discarded.

RNA was precipitated by adding 500 μL of cold isopropanol for each mL of TRIzol. The samples were incubated overnight at −20 °C and then centrifuged at 14,000× *g* for 10 min at 4 °C. The supernatant was removed, and the RNA pellet was washed twice with 75% ethanol. The samples were allowed to dry at room temperature, and the RNA was resuspended in nuclease-free water. RNA concentration and purity were determined by spectrophotometry using a NanoDrop™ (Thermo Fisher Scientific). Only samples with an A260/A280 ratio between 1.8 and 2.0 were used for subsequent analyses. The samples were stored at −80 °C until used in reverse transcription and real-time PCR.

### 2.9. Reverse Transcription and Real-Time PCR (RT-qPCR)

Before complementary DNA (cDNA) synthesis, RNA samples were treated with DNase to remove potential genomic DNA contamination. Subsequently, reverse transcription was performed using the SuperScript™ One-Step RT-PCR System with Platinum™ Taq DNA Polymerase (Thermo Fisher Scientific, Waltham, MA, USA), according to the manufacturer’s instructions. Gene expression of the 5-HT3A serotonergic receptor (*Htr3a*) was determined using quantitative real-time PCR with a StepOnePlus™ Real-Time PCR System (Applied Biosystems, Thermo Fisher Scientific, Waltham, MA, USA). Each reaction was carried out in a final volume of 20 μL, containing 10 μL of RT^2^ SYBR Green qPCR Master Mix (QIAGEN, Hilden, Alemania), 2 μL of specific primers (forward and reverse), 2 μL of cDNA, and nuclease-free water to reach the final volume. The amplification conditions consisted of an initial denaturation at 95 °C for 10 min, followed by 45 cycles of denaturation at 95 °C for 15 s and annealing/extension at 55 °C for 60 s. All samples were analyzed in triplicate. The specificity of the amplification was verified by analyzing the melting curves at the end of each run. The relative expression of the 5-HT3A gene was calculated using the 2^−ΔΔCt^ method, employing Rpl27 as an endogenous reference gene. The values obtained were normalized relative to the control group to determine the relative changes in gene expression between the experimental groups. Primer sequences used were as follows: *Htr3a* Forward 5′-TGGGTCAGAAGGGAAGG-3′, Reverse 5′-GATGATAAAGATGAGGAGGGT-3′; Rpl27 Forward 5′-AAAGCCGTCATCGTGAAGAAC-3′, Reverse 5′-GCTGTCACTTTCCGGGGATAG-3′. Primers were used at a concentration of 200 nM, with reaction efficiencies ranging between 95% and 102% derived from standard curves. Rpl27 threshold cycle (Ct) values showed no statistically significant variation across experimental groups or hemispheres (*p* > 0.05), confirming its stability as an endogenous control in this experimental context. It is clarified that the exact groups processed for molecular analysis were (n = 6/group for saline, formalin, SHAM + serum, 6-OHDA + serum, and 6-OHDA + formalin).

### 2.10. Statistical Analysis

Data were expressed as mean ± standard error of the mean (SEM). Data distribution was assessed using the Shapiro–Wilk normality test. Because some variables did not meet the normality assumption, between-group comparisons of nociceptive behavior were performed using the nonparametric Kruskal–Wallis’s test, followed by Dunn’s post hoc test for multiple comparisons. Gene expression data obtained by RT-qPCR were expressed as mean ± standard deviation (SD). Differences between experimental groups were evaluated using a one-way analysis of variance (ANOVA), followed by Tukey’s post hoc test for multiple comparisons. Time-course nociceptive behavior was analyzed using a two-way repeated-measures ANOVA (or mixed-effects model) with Group as a between-subject factor and Time as a within-subject factor, assessing main effects and Group × Time interactions (F (55,330) = 8.42, *p* < 0.0001, η^2^ = 0.38). Hemispheric differences (Left vs. Right thalamus) were evaluated using two-way ANOVA (Group × Hemisphere interaction: F (5,60) = 11.84, *p* < 0.0001, η^2^ = 0.25) with post hoc Tukey adjustments. Exact *p*-values, F-statistics, confidence intervals (95% CI), and effect sizes (eta squared, η^2^) are reported. Statistically confirming the significant difference between ipsilateral and contralateral reductions. The value of *p* < 0.05 was considered statistically significant. The analyses were performed using GraphPad Prism 8 software (GraphPad Software, San Diego, CA, USA).

## 3. Results

### 3.1. Time Course of Nociceptive Behavior During the Formalin Test

**Figure 1:**
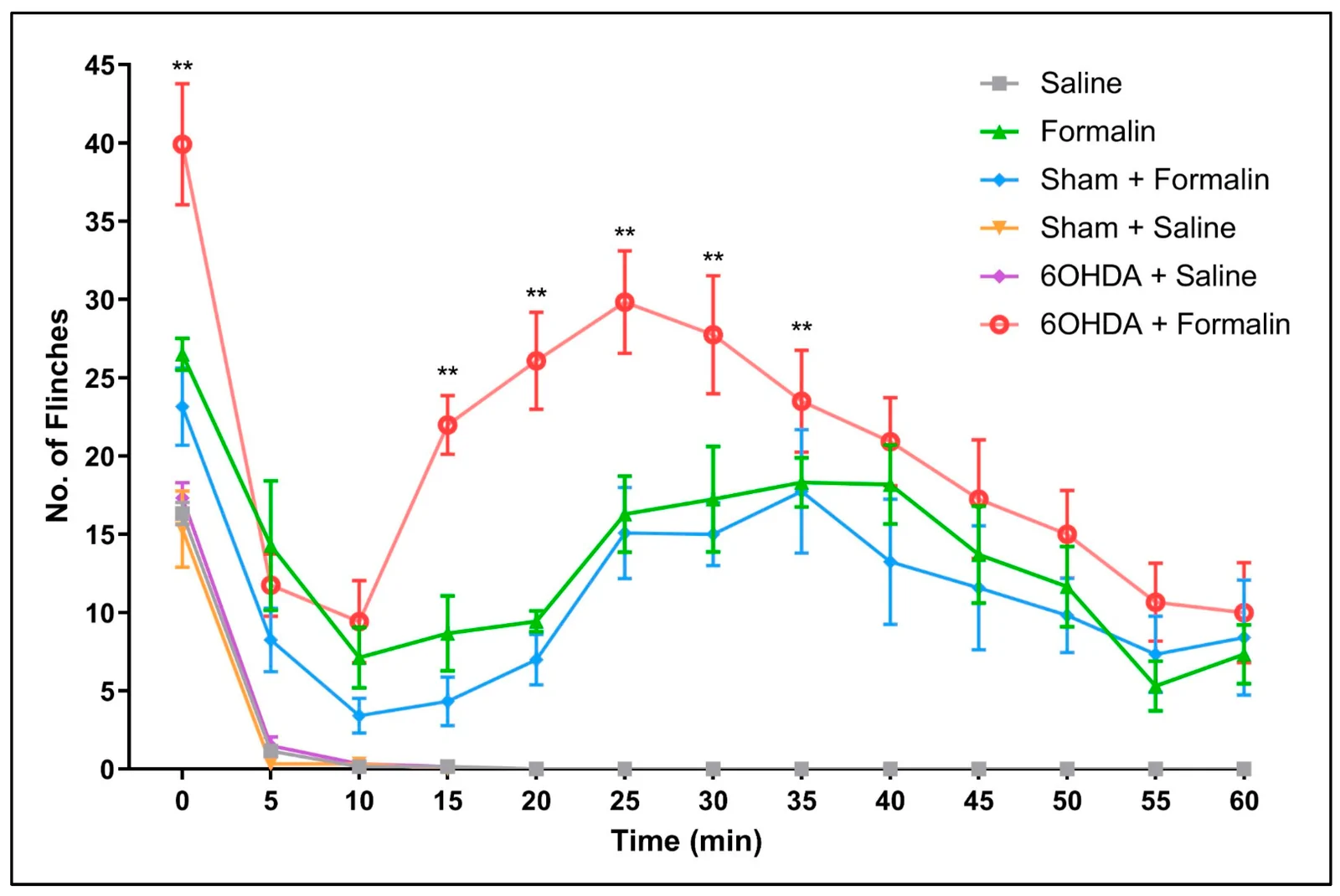
Time course of formalin-induced nociceptive behavior in rats with unilateral dopaminergic lesion induced by 6-hydroxydopamine (6-OHDA); (n = 6 per group); error bars represent Mean ± SD. Statistical analysis was performed using a two-way repeated-measures ANOVA, followed by Tukey’s post hoc test ** *p* < 0.01.

The temporal analysis of nociceptive behavior revealed significant differences among the experimental groups throughout the evaluation period (Figure 1). The groups treated with saline solution (Saline solution, SHAM + saline solution, and 6-OHDA + saline solution) exhibited a transient response during the first few minutes after injection, which virtually disappeared by minute 15 and remained near zero for the remainder of the experiment. In contrast, the groups exposed to formalin developed the biphasic response characteristic of this model of persistent pain.

Statistical analysis revealed significant differences among the treatments over time. The 6-OHDA + formalin group exhibited a significantly higher number of flinches than the other experimental groups at minutes 0, 15, 20, 25, 30, and 35 (** *p* < 0.01). During this interval, the highest level of pain-related behavior was observed, corresponding mainly to the persistent phase of the formalin test. Subsequently, nociceptive behavior gradually decreased in all formalin-treated groups until the end of the observation period.

**Figure 2:**
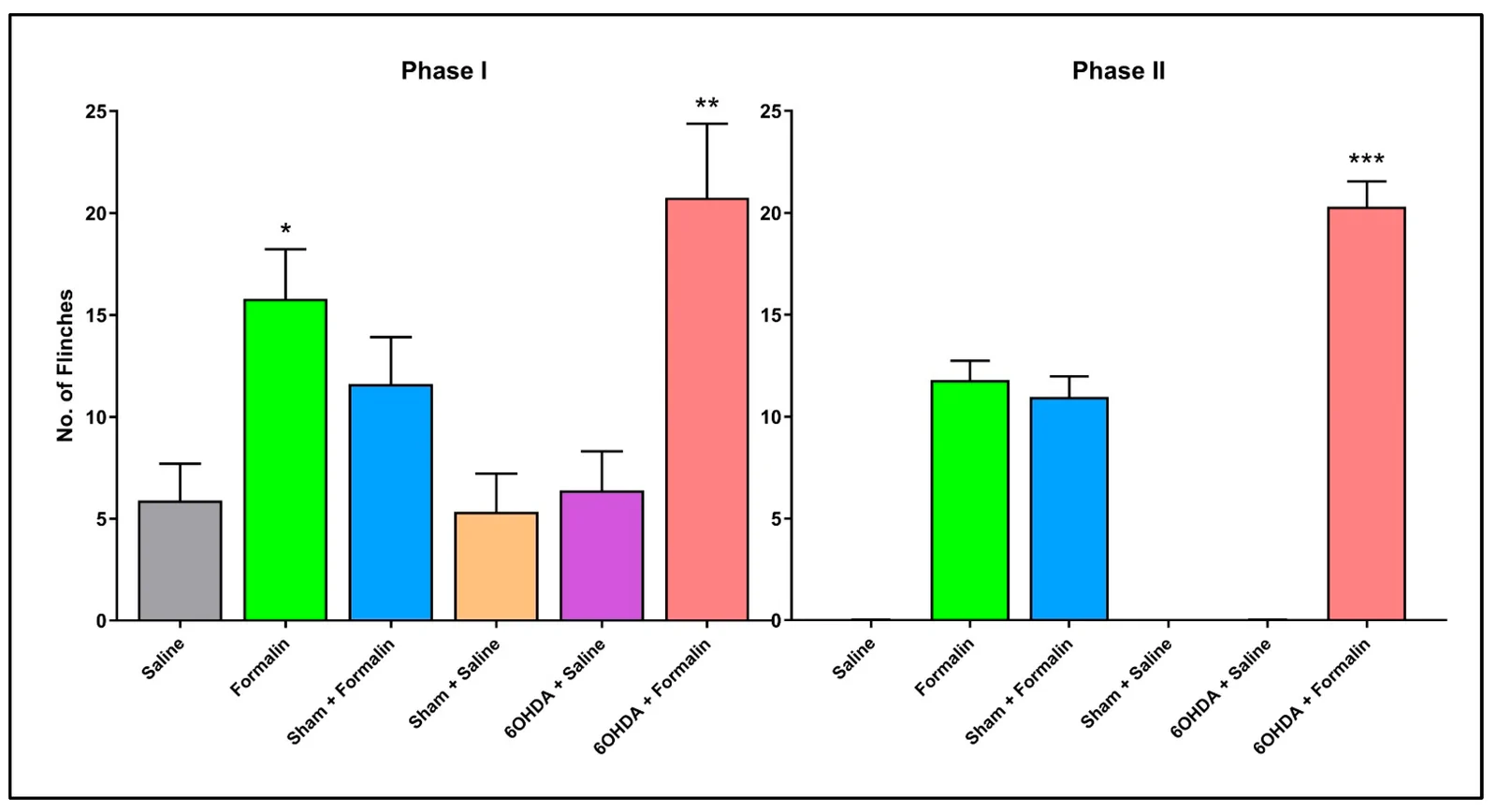
Analysis of nociceptive behavior during the acute and persistent phases of the formalin test; (n = 6 per group); error bars represent Mean ± SD. Statistical analysis was performed using two-way ANOVA, followed by Tukey’s post hoc test (* *p* < 0.05, ** *p* < 0.01, *** *p* < 0.001).

To determine whether dopaminergic lesions differentially affected the stages of the formalin-induced nociceptive response, the acute (0–15 min) and persistent (15–60 min) phases of the test were analyzed separately (Figure 2).

During the acute phase (Figure 2, left), the groups exposed to formalin exhibited more flinches than those treated with saline. The formalin group showed a significant increase in nociceptive behavior (* *p* < 0.05). Meanwhile, the 6-OHDA + formalin group showed the highest response in this phase (** *p* < 0.01). However, the magnitude of the response observed in the formalin, SHAM + formalin, and 6-OHDA + formalin groups indicates that the administration of formalin was sufficient to induce an acute nociceptive response regardless of the prior experimental condition.

In the persistent phase of the test (Figure 2, right), the saline-treated groups did not exhibit any detectable nociceptive behavior; therefore, the analysis focused on the formalin-exposed groups. In this stage, the 6-OHDA + formalin group exhibited the highest number of recorded flinches during the evaluation period (*** *p* < 0.001), clearly exceeding the responses observed in the Formalin and SHAM + Formalin groups. These latter groups showed values that were comparable to one another. The data indicate that a 6-OHDA-induced dopaminergic lesion primarily increases the nociceptive response during the persistent phase of formalin-induced pain.

Formalin time-course behavior was re-analyzed using a two-way repeated-measures ANOVA (Group × Time interaction: F(55,330) = 8.42, *p* < 0.0001, η^2^ = 0.38). Hemispheric differences in *Htr3a* expression were evaluated using a two-way ANOVA (Group × Hemisphere interaction: F(4,50) = 12.15, *p* < 0.0001, η^2^ = 0.24), statistically confirming that the ipsilateral downregulation in 6-OHDA rats is significantly greater than the contralateral reduction (*p* < 0.001).

### 3.2. Thalamic Expression of the Htr3a Gene in Hemi-Parkinsonian Rats Subjected to a Model of Persistent Formalin-Induced Pain

**Figure 3:**
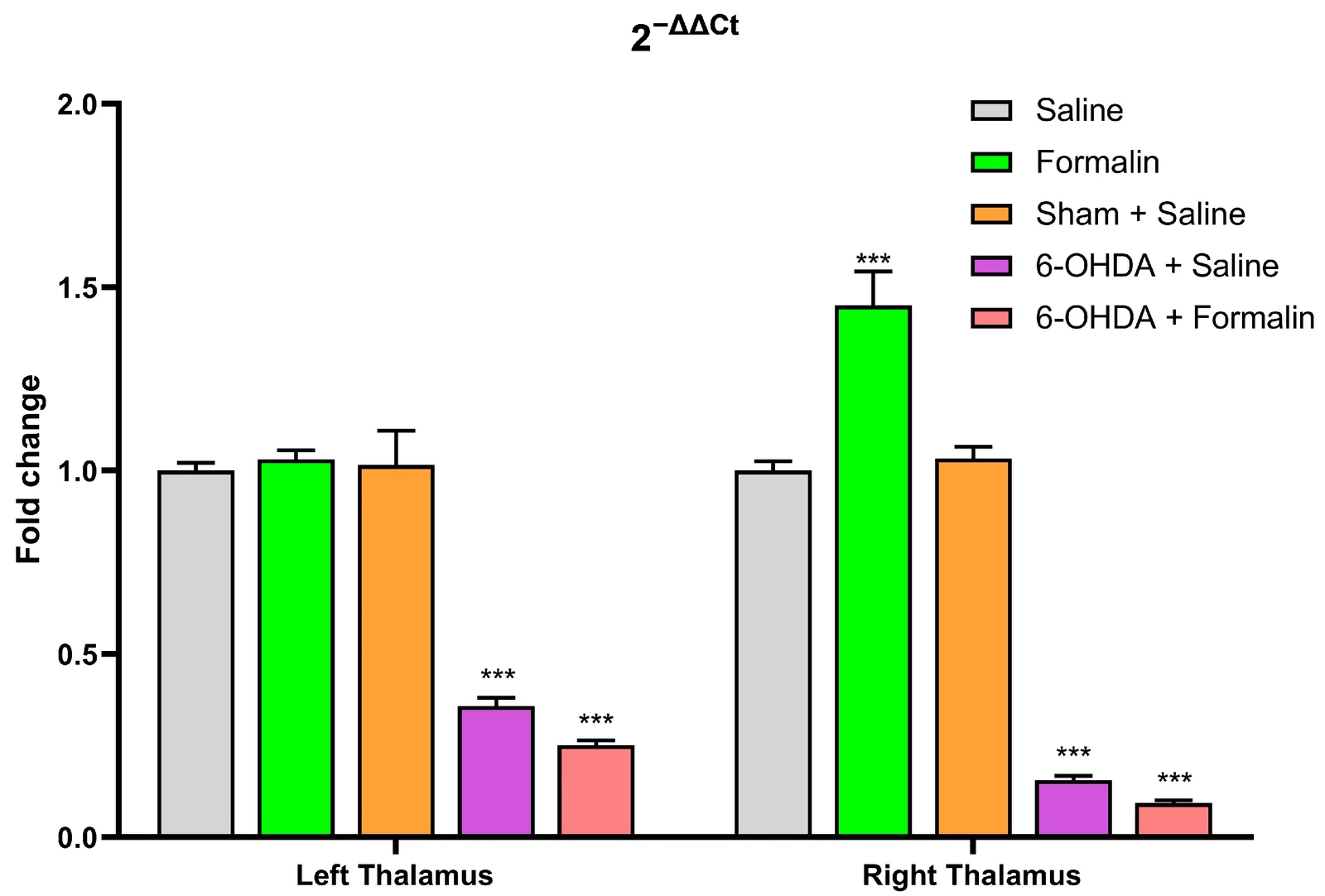
Relative expression of the *Htr3a* gene in the thalamus of rats subjected to 6-hydroxydopamine (6-OHDA)-induced dopaminergic injury and formalin-induced persistent pain; Data are expressed as mean ± SD (n = 6 per group); differences between groups were analyzed using One-way ANOVA followed by Tukey’s multiple comparison test (*** *p* < 0.001).

Relative *Htr3a* mRNA expression exhibited significant region- and treatment-dependent alterations (Figure 3). In the left thalamus (contralateral to the 6-OHDA lesion), the Saline, Formalin, and SHAM + Saline groups showed equivalent expression levels close to baseline (1.0-fold change). In contrast, 6-OHDA + Saline and 6-OHDA + Formalin groups exhibited significant downregulations in *Htr3a* expression to approximately 35% and 25% of baseline control values, respectively (** *p* < 0.001). In the right thalamus (ipsilateral to the 6-OHDA lesion), a significant transient upregulation was observed in the Formalin group (approximately 1.45-fold change vs. Saline, *** *p* < 0.001). However, 6-OHDA-lesioned groups (6-OHDA + Saline and 6-OHDA + Formalin) displayed a severe reduction in *Htr3a* mRNA, dropping below 15% and 10% of control values (** *p* < 0.001), indicating a significantly greater downregulation in the ipsilateral hemisphere compared to the contralateral thalamus (Interactions Group × Hemisphere, *p* < 0.01).

## 4. Discussion

The present study shows that unilateral dopaminergic lesion induced by 6-OHDA increased nociceptive behavior, particularly during the persistent phase of the formalin test, and was associated with a marked decrease in the relative expression of *Htr3a* mRNA in both thalamic hemispheres. The reduction was more pronounced in the thalamus ipsilateral to the lesion. Taken together, these results suggest that dopaminergic degeneration alters both central pain processing and the molecular regulation of the serotonergic system in the thalamus. The increased response during the second phase of the formalin test is particularly relevant because this stage depends not only on the initial peripheral activation of nociceptors but also on sustained inflammation, synaptic facilitation, and central sensitization. The observed hypersensitivity is consistent with findings in 6-OHDA-induced Parkinson’s models. Li et al. [17] demonstrated that a bilateral dopaminergic lesion produced mechanical and thermal hypersensitivity, accompanied by hyperexcitability of dorsal horn neurons. Accordingly, Grivet et al. [15] observed bilateral mechanical hypersensitivity, increased spinal nociceptive potentials, and greater responses in wide dynamic range neurons in hemi-Parkinsonian mice. These data support the hypothesis that dopaminergic loss reorganizes central nociceptive networks and increases sensory gain, beyond the motor alterations characteristic of the model.

Recent evidence identifies the descending serotonergic system as a key contributor to this facilitation. Following a 6-OHDA-induced lesion, increased 5-HT in the spinal cord, hyperactivity of serotonergic neurons in the nucleus raphe magnus, and greater excitability of these cells have been reported [15]. Inhibiting descending serotonergic projections reduced both mechanical hypersensitivity and spinal hyperexcitability. Furthermore, intrathecal blockade of 5-HT3 with granisetron elevated the mechanical threshold, and combined inhibition of 5-HT3 and 5-HT2A normalized the response to values close to controls [15]. This work provides mechanistic evidence that dopaminergic degeneration can convert serotonergic activity into a pain-facilitating signal.

Likewise, Patel and Dickenson [24] demonstrated that spinal *Htr3a* gene expression exerts tonic facilitation of noxious mechanical responses and that, following nerve injury, this influence extends to lower-intensity mechanical stimuli and noxious heat. Therefore, the pronociceptive function of 5-HT3 in sensitized states can increase without necessarily requiring receptor overexpression, since facilitation can also depend on greater serotonin release, changes in intracellular coupling, or plasticity of descending circuits.

However, the role of 5-HT3 is not uniform. Sawynok and Reid [25] reported that serotonin action in the spinal cord depends on the receptor subtype, the pre- or postsynaptic site, and the nociceptive paradigm, with both facilitatory and inhibitory actions. Fukushima et al. [26] later showed that 5-HT3 activation can increase inhibitory GABAergic transmission in the superficial dorsal horn. In contrast, accumulated evidence on inflammatory, neuropathic, and parkinsonian pain indicates a predominantly facilitatory effect in certain circuits. This duality indicates that the functional consequence of activating 5-HT3 depends on the cell population expressing the receptor and its position within the nociceptive network, and thus it is not appropriate to directly extrapolate spinal effects to the thalamus.

The thalamic decrease in the *Htr3a* gene expression observed in our study must therefore be distinguished from the functional activity of 5-HT3 described in the spinal cord and brainstem, because mRNA is only one of the levels determining receptor availability. Huang et al. [27] demonstrated that *Htr3a* gene expression stability can be regulated post-transcriptionally through m^6^A modifications: increased ALKBH5 stabilized the mRNA, increased 5-HT3A protein expression, enhanced receptor currents, and promoted hyperexcitability and allodynia. Although this mechanism was identified in trigeminal ganglion neurons and not in the thalamus, it shows that *Htr3a* gene expression variations can depend on both transcription and mRNA degradation, and that the correspondence among mRNA, protein, and function must be experimentally demonstrated in each tissue.

Comparison with Kwan et al. [28] reinforces this caution. These authors reported increased specific binding of the [^3^H]GR65630 radioligand in the subthalamic nucleus and, in animals with severe L-DOPA-induced dyskinesia, in the entopeduncular nucleus and the ipsilateral VA/VL thalamus. This finding is not directly contradictory to our observed reduction in the 5-HT3A receptor, because Kwan et al. assessed binding-site availability in a dyskinesia model, whereas our study quantified mRNA in a persistent pain model without L-DOPA treatment. Furthermore, chronic L-DOPA administration induces serotonergic adaptations, and the VA/VL nuclei are motor-predominant, whereas our dissection included broader thalamic tissue. Together, both studies indicate that the 5-HT3 system undergoes regional remodeling dependent on the pathophysiological context, rather than a uniform response to dopaminergic loss.

Regional specificity is also observed in other models. Kim et al. [29] identified an increase in *Htr3a* gene expression in the ipsilateral hippocampus of hemi-parkinsonian rats using RNA-seq and RT-qPCR, whereas Huang et al. [27] reported an ipsilateral increase in *Htr3a* gene expression in the trigeminal ganglion after nerve injury. In contrast, our study detected a reduction in the thalamus. These differences likely reflect cell identity, connectivity, lesion type, timing of evaluation, and the specific function of each region. The hippocampus primarily supports cognition and synaptic plasticity; the trigeminal ganglion contains primary sensory neurons; and the thalamus integrates somatosensory, affective, and motor information. Consequently, opposing changes in *Htr3a* gene expression should be interpreted as regionally differentiated responses rather than mutually exclusive results.

The functional anatomy of the thalamus supports this interpretation. Herrero et al. [30] described the thalamus as a heterogeneous set of nuclei that select, amplify, and integrate sensory and motor signals via reciprocal connections with the cortex and basal ganglia. The functional relevance of 5-HT3 in the thalamus also appears to depend on the nucleus. Xiao et al. [31] showed that the 5-HT3 receptor antagonist LY-278,584 did not modify serotonin-induced antinociception in the submedius nucleus, whereas 5-HT1A and 5-HT2 receptors contributed to this effect. Therefore, 5-HT3 signaling may be limited within certain thalamic analgesic circuits, even when it is pronociceptive in the spinal cord or brainstem.

Within this framework, the decrease in *Htr3a* gene expression could be a homeostatic response to increased serotonergic activity in the nociceptive neuroaxis. Sustained serotonin exposure or heightened circuit excitability could downregulate the mRNA to limit ionotropic signaling. However, this explanation remains a hypothesis, as our study did not measure thalamic serotonin, mRNA stability, receptor occupancy, or neuronal excitability. An alternative interpretation is that the reduction reflects changes in tissue cellular composition, specific modifications of certain nuclei, or a secondary response to persistent pain, rather than a causal mechanism of hyperalgesia.

## 5. Limitations

Despite the findings of this study, some limitations remain. First, the dopaminergic lesion induced by 6-OHDA was functionally and behaviorally validated by the apomorphine rotation test (>7 turns/min), but was not complemented with immunohistochemical verification (e.g., tyrosine hydroxylase staining) or quantitative assays of striatal dopamine content. Therefore, the terms related to dopaminergic loss reflect functional hemi-Parkinsonism rather than direct histological quantification. Second, our study measured *Htr3a* expression only at the level of mRNA transcription by RT-qPCR. Changes in gene expression do not directly equate to alterations in the protein density of the 5-HT3A receptor; translational and epigenetic studies (such as mRNA stability or m6A modifications) can be performed in future studies. On the other hand, the absence of the SHAM + Formalin group in the molecular assay as a limitation of the study indicates that, although SHAM + Serum controls surgical artifacts, the SHAM + Serum group did not show significant differences in the expression of the *Htr3a* thalamic gene compared to the intact group (*p* > 0.05), confirming that stereotactic surgery, microinjection pressure, and vehicle delivery do not alter *Htr3a* thalamic expression on their own. Future studies could include SHAM + Formalin to evaluate possible subtle effects of the interaction between sham surgery and inflammatory pain at the transcriptional level. Finally, without direct electrophysiological or microdialysis measurements, the hypothesis we propose on homeostatic adaptation is far from a possible proposal on this mechanism. Future studies incorporating Western blot/IHC, pharmacological receptor blockades, and single-unit thalamic electrophysiology are necessary to establish causality.

## 6. Conclusions

The unilateral 6-OHDA-induced dopaminergic lesion is associated with increased persistent-phase formalin nociceptive behavior and a marked bilateral (predominantly ipsilateral) reduction in thalamic *Htr3a* mRNA expression. These findings highlight a region-specific transcriptional adaptation of the thalamic serotonergic system in experimental parkinsonian persistent pain, though further functional studies are required to establish causal mechanisms.

## Data Availability

The data presented in this study are available on request from the corresponding author due to institutional reasons.

## Abbreviations

The following abbreviations are used in this manuscript:

5-HT	5-Hydroxytryptamine
5-HT3	5-Hydroxytryptamine type 3 receptor
5-HT3A	5-Hydroxytryptamine type 3A receptor
6-OHDA	6-Hydroxydopamine
cDNA	Complementary DNA
mRNA	Messenger RNA
PCR	Polymerase chain reaction
PD	Parkinson’s disease
RT-qPCR	Reverse transcription quantitative real-time polymerase chain reaction
